# Spring Scrub Typhus, People’s Republic of China

**DOI:** 10.3201/eid1209.060257

**Published:** 2006-09

**Authors:** Min Cao, Hengbin Guo, Tang Tang, Changjun Wang, Xianfu Li, Xiuzhen Pan, Jiaqi Tang

**Affiliations:** * Research Institute for Medicine of Nanjing Command, Nanjing, People's Republic of China;; † University of Pennsylvania School of Medicine, Philadelphia, Pennsylvania, USA

**Keywords:** scrub typhus, Spring, Orientia tsutsugamushi, epidemiologic investigation, letter

**To the Editor:** Pingtan Island, in the eastern Fujian Province, People's Republic of China, has been a traditional focus of summer scrub typhus. In the early 1950s, the health of the residents was compromised by scrub typhus, with an incidence of 1,000 cases/100,000 population and a case-fatality rate of 13.6%. With the understanding of the pathogen and application of effective treatments (*1**,**2*), the epidemic was brought under control. Documentation showed that from 1960 through 1990, the annual incidence of scrub typhus maintained a level of 50–100 cases/year. Since 1990, cases have decreased sharply.

The usual epidemic season for scrub typhus on the island was summer. The first outbreak of spring scrub typhus occurred in 2000 in the town of Beicuo; 10 cases were reported. Beicuo, population 10,000, is located in southwestern Pingtan Island. The first patient visited the local hospital on April 6, 2000, with a high fever, cough, and headache. The initial exclusion of scrub typhus because of the spring time frame made the diagnosis difficult until a typical black eschar was found on the patient's waist. This case called attention to spring scrub typhus and led to the timely diagnosis and treatment of the subsequent cases. By 2005, a total of 28 spring cases were on file. An indirect immunofluorescence antibody method with Gilliam strain antigen, as described (*3**,**4*), was applied to the above samples for serologic analysis. Samples with antibody titers >64 were considered diagnostic. All 28 case-patients were identified as having antibodies to *Orientia tsutsugamushi* 8–20 days after the onset of the disease (Table).

**Table:** Clinical manifestations of spring scrub typhus, 2000–2005, Pingtan Island, People's Republic of China

Characteristic	No. patients (%)
Male	24 (86)
Female	4 (14)
Symptom	
Fever	28 (100)
Eschar	25 (89.2)
Node	28 (100)
Rash	25 (89)
Cough	19 (68)
Headache	13 (46)
Chill	15 (53)
Fatigue	16 (57)
Myalgia	10 (35)
Vomiting	8 (29)
Serologic titer	
IgG titer of IFA,* median (range)	1,280 (320–2,560)
Age, y	
Median (range)	21.5 (7–59)

*IFA, indirect immunofluorescence assay; IgG, immunoglobulin G.

The number of cases of spring scrub typhus from 2000 to 2005 were 10, 7, 9, 0, 0, and 2, respectively. The disease was prominent in farm workers aged 40 to 49 years (10 cases). Most younger persons aged 20–40 years, had left the area for better income, so their case number was relatively low (4 cases). Five patients were military personnel, of which 4 were susceptible new recruits from various regions where scrub typhus was not found. Eight cases were associated with children who often played in the grassland and woods. The chance of getting infected with the scrub typhus agent is increased by frequent exposure to the vector mites, which inhabit areas rich in vegetation.

We performed an investigation on the possible hosts and vectors of spring scrub typhus since 2002. Rodents were trapped in April and May 2002. Of 246 captured rodents, *Rattus losea* comprised 32.5% of the collection and had a high mite-carrying rate and mite-carrying index (87.5% and 19.9%, respectively). Mites were collected from the captured rodents. Among these mites, 2,100 *Leptotrombidium deliense* accounted for 94.1% of all the mites.

*O. tsutsugamushi* was isolated by peritoneally injecting mice of KM species with the patient's untreated blood, the triturated viscera of the rats (*R. losea*), and the triturated mites (*L. deliense*). This process resulted in 3 identification of *O. tsutsugamushi* strains, which we named Ptan, Ptan2, and Ptan3 (GenBank accession nos. DQ517961, DQ517962, and DQ517963). PCR was performed as previously described (*5*), and the sequences of the gene encoding the 56-kDa protein from the 3 *O. tsutsugamushi* isolates shared >99.8% homology. They also shared 96% homology with *O. tsutsugamushi* Karp strain.

This study verified Pingtan Island as a focus of spring scrub typhus by demonstrating the existence of the pathogen among patients, a rodent host *R. losea,* and the vector *L. deliense*. As demonstrated by Yu et al. in 1953 (*6*), *R. losea* and *L. delinese* were also the host and vector of summer scrub typhus on the island. *L. delinese* formerly appeared in late May and now can be found in March. The earlier appearance of these mites might be related to the warming weather. The local meteorologic data showed that between 1953 and 1996, the average March temperature never exceeded 12.7°C. However, since 1997, temperature increases have been recorded. During 2000–2002, the average March temperatures were 13.8°C, 15.1°C, and 16.4°C, for each year, respectively. The earlier appearance of the vector mites might explain the spring cases of scrub typhus. No cases of scrub typhus were reported in 2003 and 2004. This finding might be due to the successful preventive measures, including education about scrub typhus and instructions for using personal protective gear against mite bites when working in the fields. Also in 2003, an unusually low amount of precipitation limited the growth of vegetation, which subsequently restricted the habitat of the mites.

When we compared the patients with spring and summer scrub typhus, we observed similar epidemiologic characteristics, including clinical symptoms, pathogen hosts and vectors, and epidemic pathway. The local meteorologic records confirmed an increase in average March temperature since 1997. We suspect that these reports of spring cases represent a widening of the epidemic season of summer scrub typhus because of the increase in local temperature. We plan to seek confirmation by comparing the genetic relatedness of this spring scrub typhus isolate with that of the summer isolate, serologically identified by Yu et al. (*7*) as Gilliam type.
